# Congenital contractural arachnodactyly (Beals syndrome)

**DOI:** 10.1186/1750-1172-1-20

**Published:** 2006-06-01

**Authors:** Ergül Tunçbilek, Yasemin Alanay

**Affiliations:** 1 Clinical Genetics Unit, Department of Pediatrics, Faculty of Medicine, Hacettepe University, 06100, Sihhiye, Ankara, Turkey

## Abstract

Congenital contractural arachnodactyly (Beals syndrome) is an autosomal dominantly inherited connective tissue disorder characterized by multiple flexion contractures, arachnodactyly, severe kyphoscoliosis, abnormal pinnae and muscular hypoplasia. It is caused by a mutation in *FBN2 *gene on chromosome 5q23. Although the clinical features can be similar to Marfan syndrome (MFS), multiple joint contractures (especially elbow, knee and finger joints), and crumpled ears in the absence of significant aortic root dilatation are characteristic of Beals syndrome and rarely found in Marfan syndrome. The incidence of CCA is unknown and its prevalence is difficult to estimate considering the overlap in phenotype with MFS; the number of patients reported has increased following the identification of *FBN2 *mutation. Molecular prenatal diagnosis is possible. Ultrasound imaging may be used to demonstrate joint contractures and hypokinesia in suspected cases. Management of children with CCA is symptomatic. Spontaneous improvement in camptodactyly and contractures is observed but residual camptodactyly always remains. Early intervention for scoliosis can prevent morbidity later in life. Cardiac evaluation and ophthalmologic evaluations are recommended.

## Disease name and synonyms

Congenital contractural arachnodactyly, (CCA);

Beals syndrome;

Beals-Hecht syndrome;

Arthrogryposis, distal, type 9.

## Definition

Congenital contractural arachnodactyly (CCA; Beals syndrome, MIM # 121050) is an autosomal dominantly inherited connective tissue disorder that shares phenotypical features with Marfan syndrome (MFS, MIM # 154700). Beals syndrome has distinct features however, and is caused by a mutation in the fibrillin-2 gene (*FBN2*) in 5q23, while Marfan syndrome is caused by mutations in fibrillin-1 [1].

## Differential diagnosis

The main differential diagnosis is Marfan syndrome. CCA shares skeletal features with MFS such as marfanoid habitus, arachnodactyly, camptodactyly and kyphoscoliosis. However, CCA patients have crumpled appearance of ear helix and congenital contractures, and do not typically have the ocular and cardiovascular complications seen in MFS. Although the presence of contractures is specific for CCA, molecularly proven MFS patients with mild contractures have been reported.

Lens subluxation is present in approximately half of patients with MFS and the most common cardiovascular complications are dilatation of aortic root and mitral valve prolapse. Mitral regurgitation is a well established feature of CCA. Other congenital heart defects have been reported, but aortic dilatation is mild in CCA and stationary, measurements being always less than 2SD above mean [2]. In MFS, the aortic root dilatation expands beyond 2SD and is progressive. Ectopia lentis is very rare in CCA, but general ocular complications are estimated to be present in 20% of patients with CCA [2]. The difficulty in differentiating CCA and MFS is best demonstrated by the fact that the original patient first described by Dr. Antoine Marfan in 1898 has been stated to have CCA, rather than MFS.

Patients with neonatal Marfan syndrome may have crumpled ears, blurring the differential diagnosis process. Neonatal MFS patients and severe lethal CCA share more features, such as arachnodactyly, joint contractures, and some facial features. However, the cardiac manifestations, mainly valvular problems that lead to early death in neonatal MFS and, on the other hand, the scoliosis seen in CCA are helpful in the differential diagnosis.

The overlap in clinical features has a molecular basis. CCA and MFS result from mutations in two homologous genes, *FBN2 *and *FBN1*, which are highly similar but distinct genes situated in 5q23-31 and 15q15-21.3 chromosome, respectively [3].

## Epidemiology

Despite the recognition of MFS as a syndrome for more than a century, CCA has only recently been accepted as a distinct entity. After the demonstration that CCA is linked to mutations in *FBN2*, the two syndromes were considered as truly separate entities.

The incidence of CCA is unknown and its prevalence is difficult to estimate considering the overlap in phenotype with MFS. The number of patients reported has increased following the identification of a mutation in *FBN2 *gene. The prevalence of CCA in the general population may be estimated with more realistic figures in the near feature.

## Clinical description

The individuals affected by CCA can be easily identified at birth with clenched position of hands (fist-like), and by their ears having a crumpled irregular superior helix and prominent antihelix and root of helix; the fingers are long and there is elongation of phalanges on X-rays. The auricular shape may become milder and camptodactyly may partially resolve spontaneously [2-4]. Pectus carinatum, striae and highly arched palate are additional features.

Contractures of varying degrees at birth, mainly involving the large joints, are present in all affected children. Elbows, knees and fingers are most commonly involved. The contractures may be mild and tend to reduce in severity, but residual camptodactyly always remains present. The arm span exceeds body height but the discrepancy may be underestimated due to contractures of elbows and fingers. The same holds for the lower body portion with knee contractures. The most serious complication in CCA is scoliosis and sometimes kyphoscoliosis mandating surgery.

There has been increasing number of reports demonstrating cardiovascular involvement in CCA patients with molecularly proven *FBN2 *mutations. Mitral valve prolapse can be seen among patients with *FBN2 *mutations. It has been previously suggested that congenital heart defects are relatively common, but only 2/22 CCA mutation-positive patients have been reported to have a heart defect [3].

Ophthalmologic abnormalities, including heterotopia are reported in 20% of cases. Ectopia lentis is very rare. In 2004, two cases, one with blue sclerae and glaucomatous optic disc cupping, and another with partial coloboma of the lens, mild cataract, abnormal cilliary body and glaucoma were reported [5].

Individuals with CCA are expected to be mentally normal. Delay in the motor development is common due to contractures. Dislocation of joints, especially patellae has been reported. In the past, linkage studies have failed to demonstrate a collagen type I defect. Generalized osteoporosis is well documented, but fractures of long bones are uncommon. A case report with a femoral fracture in a neonate discussed a predisposition versus birth injury due to cesarean delivery [6].

A single report described a mother and daughter with classic and severe lethal CCA, respectively [7]. The *FBN2 *mutation resulted in the identical missplicing of exon 34 in both patients. The mother was a somatic mosaic for the mutation and demonstrated the classic CCA phenotype. However, the presence of developmental cardiac abnormalities as septal defects, interrupted aortic arch and single umbilical artery in addition to duodenal atresia, esophageal atresia, and intestinal malrotation in the daughter, raises the question of a co-existing VACTERL-like (vertebral anomalies, anal atresia, cardiovascular malformations, tracheoesophageal fistula, renal and limb anomalies) entity. Therefore, when the diagnosis is uncertain and not molecularly proven, an unrelated second entity should be considered in the presence of such unusual features in addition to the classic CCA phenotype.

## Management

Management of children with CCA is symptomatic. Camptodactyly and contractures involving other joints may resolve with time, but residual camptodactyly always remains. The growing child should be followed for deformities in the axial skeleton. Kyphoscoliosis is often present at birth or in early childhood. Routine physical examination for spinal deformity and early intervention for scoliosis can prevent morbidity later in life.

An initial cardiac evaluation with serial echocardiography is indicated if abnormal findings are present. Although ocular involvement is yet unclear, a thorough ophthalmologic evaluation is recommended.

There is no evidence of shortened lifespan. Individuals with CCA are to live normal lives unless complicated with cardiac problems or severe deformity of the vertebrae.

## Etiology

CCA is an autosomal dominantly inherited single gene disorder, first described by Beals and Hecht in 1971 [4]. It is caused by a mutation in *FBN2 *gene on chromosome 5q23. This second fibrillin gene was discovered during research efforts to identify the genetic basis of MFS. The so far identified mutations in *FBN2 *cluster in a limited region similar to where severe MFS cluster in *FBN1*, between exons 23 and 34, the so-called "neonatal region". Therefore, mutations clustered in the region of *FBN2 *are homologous to those in *FBN1 *related to neonatal severe MFS. The mutation related to skipping of exon 34 of *FBN2 *specifically causes the severe/lethal CCA phenotype [1].

Fibrillins are large, cysteine-rich glycoproteins forming microfibrils. They play a central role in elastic fibrillogenesis. It is hypothesized that the expression of fibrillin-2 directs the assembly of elastic fibers during early embryogenesis, while fibrillin-1 provides the major structural force bearing support in many tissues and organs. Identified mutations in the *FBN2 *gene change the structure of the central region of the fibrillin-2 protein by altering a cysteine, deleting an entire domain or potentially adding a novel glycosylation site to the domain. In contrast, missense mutations altering aminoacids in the calcium-binding consensus sequence in EGF-like domain is the main underlying molecular defect in MFS related to fibrillin-1 protein [1].

Parental somatic and germline mosaicism have been observed in families with affected siblings with CCA [8]. The presence of gonadal mosaicism in the *FBN2 *gene is important for accurate genetic counseling. Somatic mosaicism is thought to be the explanation for the phenotypical variations in severity among affected individuals.

Mutations in the *FBN2 *gene have been identified in almost half of the CCA cases. It is now a challenge to investigate the mutations outside the "neonatal region" which might be the molecular explanation for some cases with Marfan-like phenotype.

## Genetic counseling

CCA is an autosomal dominantly inherited disorder. The recurrence rate is estimated to be 50%. Germline mosaicism should always be kept in mind when counseling families with sporadic cases. If neither parent is clinically affected, there is still a small (but unknown) risk to the sibs because germline mosaicism has been reported in three unrelated families; in one case, an unaffected father had two children with CCA [8]. Furthermore, parents should always be evaluated along with the patient considering the possibility of somatic mosaicism leading to a milder phenotype in the parent with typical features of CCA in the offspring.

## Antenatal diagnosis

Molecular antenatal diagnosis is possible if indicated and desired by the parents after appropriate genetic counseling. Ultrasound imaging may be used to demonstrate joint contractures and hypokinesia in suspected cases.
